# Biosynthesis of glucocorticoids in tumors

**DOI:** 10.1172/JCI174686

**Published:** 2023-11-01

**Authors:** Nicola Cirillo

**Affiliations:** Faculty of Medicine, Dentistry and Health Sciences, University of Melbourne, Melbourne, Victoria, Australia.

**Keywords:** Endocrinology, Oncology, Cancer

**To the Editor:** The study by Taves et al. (1) provides evidence that tumor-derived glucocorticoids suppress local antitumor immunity, thereby promoting tumor growth in mice. In one of the pilot experiments informing the design of the study, corticosterone could not be detected in the supernatant of malignant mouse cell lines following incubation with 11-deoxycorticosterone (DOC), the immediate precursor of corticosterone, thus supposedly indicating the absence of constitutive CYP11B1 activity. The authors concluded that tumor cells are not capable of synthesizing glucocorticoids de novo and that the primary pathway for the production of intratumor corticosterone is the recycling of inactive metabolites via 11β-HSD1. This is in sharp contrast to current evidence showing that primary synthesis of steroids is a common occurrence in nonadrenal tissues, including cancers (recently reviewed in ref. 2).

Determining the origin of tumor-derived steroids is not trivial. If the steroid is produced exclusively by a recycling pathway, the bioavailability of glucocorticoid metabolites in the tumor microenvironment would be the key driving (or limiting) factor regulating local steroid production. This has salient clinical implications, as glucocorticoids are used in patients with cancer as first-line treatment and as adjuvants. Additionally, several potentially malignant disorders are treated with synthetic glucocorticoids, and this poses potential risks, not only in terms of systemic immune suppression, but also because this treatment may promote tumor growth by supplying steroid metabolites. If, however, glucocorticoids were produced by tumor cells de novo, the availability of usable metabolites would not be pivotal, and, in turn, the potential translatability of 11β-HSD1 inhibitors in cancer treatment would be markedly hampered.

Despite presenting a clear statement in the title, the study by Taves et al. (1) fails to provide definitive evidence that mouse tumor cells are unable to synthesize corticosterone de novo. The crucial point in my reasoning is the appreciation that the steroid biosynthetic pathway differs between mammals and rodents, and these differences may have been overlooked by the authors when interpreting the results.

In humans, conversion of progesterone and 17α-hydroxyprogesterone to DOC (mineralocorticoid pathway) and 11-deoxycortisol (glucocorticoid pathway) leads to the production of aldosterone (via CYP11B2) and cortisol (via CYP11B1), respectively. These pathways are both biochemically and spatially distinct, a concept referred to as “functional zonation.” Conversely, rodents do not produce cortisol, and the corticosterone synthesized in the adrenal glands may be both end product and intermediate of this biosynthetic pathway (Figure 1). The restricted synthesis of aldosterone in the zona glomerulosa and of corticosterone in the zona fasciculata is a result of the adrenal expression of CYP11B2 predominantly in the zona glomerulosa for aldosterone synthesis and the expression of the 11β-hydroxylase (CYP11B1) in the zona fasciculata for corticosterone production.

In nonadrenal tissues, however, there is no spatial segregation (or functional zonation), and both isozymes are expressed in the same cells. Therefore, incubation with DOC may result in the predominant production of either corticosterone or aldosterone based on DOC conversion kinetics and *Cyp11b* expression profile. Hence, it is possible that, in the study by Taves et al. (1), DOC was converted to aldosterone by CYP11B2 and little free corticosterone was present in the supernatants within 24 hours.

The existence of this common pathway also explains why glucocorticoid production does not rely exclusively on CYP11B1 activity in rodents. *Cyp11b1*-null mice produce detectable levels of corticosterone, the concentration of which increases in response to glucose challenge (3), confirming that CYP11B2 has 11β-hydroxylase activity and hence can synthesize corticosterone. Assessment of corticosterone levels following incubation with DOC may not therefore be a surrogate for the presence of a functional CYP11B1 cytochrome.

The assay used for detection of corticosterone in culture supernatants is also important. The authors chose ELISA to measure corticosterone levels, and this may not be the most accurate method. Nonadrenal tissue-derived corticosterone and steroid metabolites have been successfully detected in culture supernatants in vitro and ex vivo using more sensitive techniques, such as radioimmunoassay (4) and radiometric conversion assays (5).

After consideration of the findings of the previous studies, I conclude that current research solidly demonstrates that tumors produce glucocorticoids de novo and that the study by Taves et al. (1) does not contradict this evidence.

## Figures and Tables

**Figure 1:**
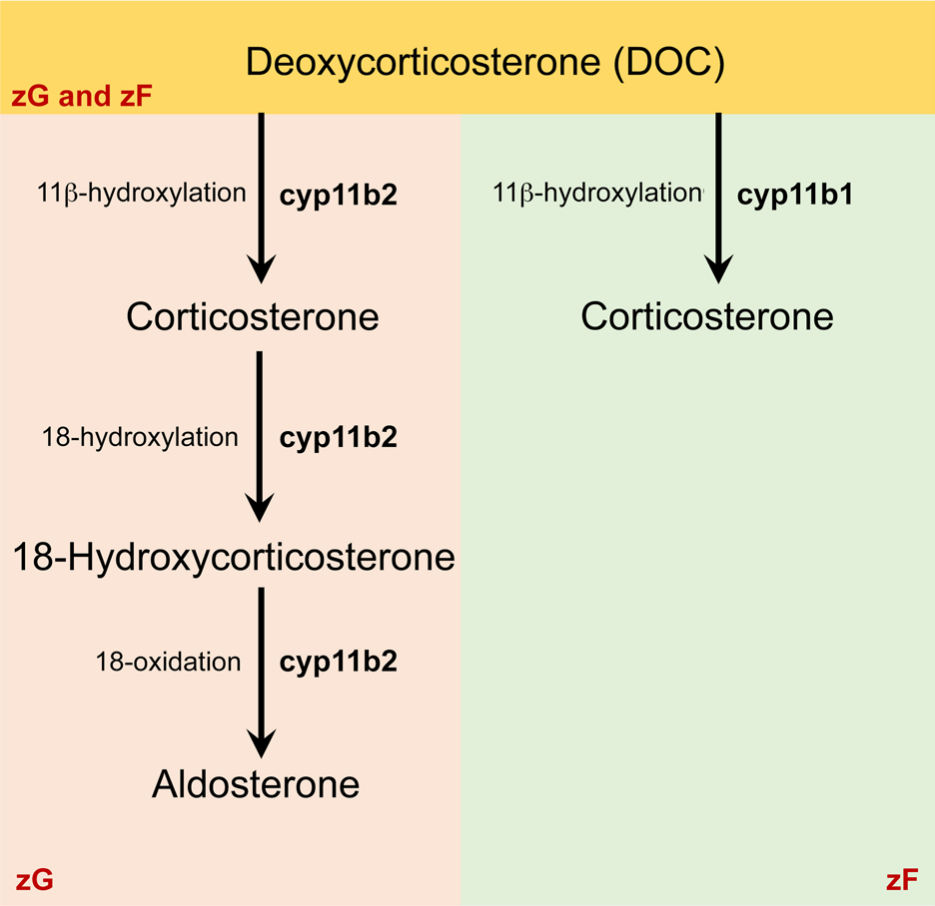
Main biosynthetic pathways of corticosteroids in mice. In mice, like in humans, aldosterone biosynthesis is restricted to the zona glomerulosa. Unlike that in humans, the major glucocorticoid synthesized in mice is corticosterone (instead of cortisol), which is produced from the same precursor deoxycorticosterone by 11β-hydroxylase (CYP11B1) in the zona fasciculata (zF). Therefore, in mice, corticosterone is both an intermediate (zona glomerulosa [zG]) and end product (zF) of steroid biosynthesis.

## Version 1 11/01/2023

Electronic publication
